# Exploration of plasma metabolite levels in healthy nursery pigs in response to environmental enrichment and disease resilience

**DOI:** 10.1093/jas/skad033

**Published:** 2023-01-27

**Authors:** Elda Dervishi, Xuechun Bai, Jian Cheng, Frederic Fortin, Mike K Dyck, John C S Harding, Yolande M Seddon, Jack C M Dekkers, PigGen Canada, Graham Plastow

**Affiliations:** Livestock Gentec, Department of Agriculture, Food and Nutritional Science, University of Alberta, Edmonton, AB T6G 2R3, Canada; Livestock Gentec, Department of Agriculture, Food and Nutritional Science, University of Alberta, Edmonton, AB T6G 2R3, Canada; Department of Animal Science, Iowa State University, Ames, IA 50011, USA; Centre de developpement du porc du Quebec inc. (CDPQ), Quebec City, QC, Canada; Livestock Gentec, Department of Agriculture, Food and Nutritional Science, University of Alberta, Edmonton, AB T6G 2R3, Canada; Department of Large Animal Clinical Sciences, University of Saskatchewan, Saskatoon, SK, Canada; Department of Large Animal Clinical Sciences, University of Saskatchewan, Saskatoon, SK, Canada; Department of Animal Science, Iowa State University, Ames, IA 50011, USA; PigGen Canada Research Consortium, Guelph, ON N1H4G8Canada; Livestock Gentec, Department of Agriculture, Food and Nutritional Science, University of Alberta, Edmonton, AB T6G 2R3, Canada

**Keywords:** disease challenge, enrichment, metabolites, pig, resilience

## Abstract

The purpose of this study was to explore plasma metabolite levels in young healthy pigs and their potential association with disease resilience and estimate genetic and phenotypic correlation with the change in lymphocyte concentration following disease challenge. Plasma samples were collected from 968 healthy nursery pigs over 15 batches at an average of 28 ± 3.23 d of age. Forty-four metabolites were identified and quantified by nuclear magnetic resonance. Pigs were then introduced into a natural disease challenge barn, and were classified into four groups based on the growth rate of each animal in the grow-to-finish phase (GFGR) and treatment rate (TR): resilient (RES), average (MID), susceptible (SUS), and dead (pigs that died before harvest). Blood samples were collected from all pigs before and 2 wk after disease challenge and complete blood count was determined. Environmental enrichment (inedible point source objects) was provided for half of the pigs in seven batches (*N* = 205) to evaluate its impact on resilience and metabolite concentrations. Concentration of all metabolites was affected by batch, while entry age affected the concentration of 16 metabolites. The concentration of creatinine was significantly lower for pigs classified as “dead” and “susceptible” when compared to “average” (*P* < 0.05). Pigs that received enrichment had significantly lower concentrations of six metabolites compared with pigs that did not receive enrichment (*P* ≤ 0.05). Both, group classification and enrichment affected metabolites that are involved in the same pathways of valine, leucine, and isoleucine biosynthesis and degradation. Resilient pigs had higher increase in lymphocyte concentration after disease challenge. The concentration of plasma l-α-aminobutyric acid was significantly negatively genetically correlated with the change in lymphocyte concentration following challenge. In conclusion, creatinine concentration in healthy nursery pigs was lower in pigs classified as susceptible or dead after disease challenge, whilst l-α-aminobutyric may be a genetic biomarker of lymphocyte response after pathogen exposure, and both deserve further investigation. Batch, entry age, and environmental enrichment were important factors affecting the concentration of metabolites and should be taken into consideration in future studies.

## Teaser

This study identifies l-α-aminobutyric acid as potential genetic marker for the magnitude of lymphocyte response after pathogen exposure. In addition, this study contributes to understanding the impact of environmental enrichment on plasma metabolites concentration.

## Introduction

Swine production in N. America and Europe is typically organized as a pyramid, where genetic nucleus farms owned by breeding companies create genetic improvement at the top and commercial farms produce slaughter pigs at the bottom of the pyramid (Er et al., 2016). The pigs in the nucleus farms are maintained in healthier and biosecure environments to provide the opportunity for expression of genetic potential compared to the commercial farms, where the level of biosecurity is harder to maintain and pathogens tend to be present (Bai and Plastow, 2022). However, in practice only 40%–70% of the genetic improvement realized in the nucleus will result in improved performance at the commercial level (Meuwissen et al., 2016). This gap in performance is due to the impact of environmental stressors, nutrition, and disease, preventing pigs from reaching their genetic potential for growth under commercial conditions. Typically pigs on commercial farms are faced with a wide range of stresses and challenges, for example, weaning, mixing, food competition, aggression, poor indoor air quality, temperature stress, transportation, as well as disease. Often, pigs are reared in a barren environment, which may lead to chronic stress, which in turn negatively impacts immune response (Bolhuis et al., 2005).

Resilience in pigs has been described as the capacity of the animal to be minimally affected by disturbances such as stress or disease and to rapidly return to the state experienced prior to onset of the disturbance (Colditz and Hine, 2016; Berghof et al., 2019). Improving resilience can be achieved using genetic selection and/or environmental modifications (Mulder and Rashidi, 2017; Bai et al., 2020; Bai and Plastow, 2022). There is also an increased interest from consumers that pigs are raised with high health and welfare. This, in part, can help to reduce the use of antibiotics on pig farms and the associated increase in antimicrobial resistance (Bai and Plastow, 2022). It is expected that, when challenged by disease, resilient pigs will grow better and will return to health more quickly than less resilient pigs, in turn requiring less antimicrobial use. In addition, management that supports resilience and reduces the impact of disease on commercial farms will have a positive impact, providing improved performance and reducing the reliance on antimicrobial treatments.

There is mounting evidence that environmental enrichment modifies pig behavior, stress, and immune response (Bolhuis et al., 2005; Fàbrega et al., 2019; Reimert et al. 2014a, 2014b; Luo et al., 2020). Providing environmental enrichment increases brain-derived neurotrophic factor (BDNF) in the serum (Rault el al., 2018) and the abundance of proteins related to protein synthesis in the hippocampus (Arroyo et al., 2020). In addition, providing enrichment alters the concentration of blood metabolites in pigs (Fàbrega et al., 2019; Dervishi et al., 2021a). Different categories of enrichments exists for pigs including: social, occupational, physical, sensory, and nutritional enrichments (van de Weerd and Day, 2009). Point-source environmental enrichment (example: a suspended piece of rope or jute sack) are limited in size and restricted to a single location in the pen (van de Weerd and Day, 2009). van Dixhoorn et al. (2016) showed a direct link between environmental enrichment and clinical outcome in pigs infected with porcine reproductive and respiratory syndrome virus (PRRSV). Previous studies have used physical or edible enrichment for pigs such as straw, wood shavings, moist peat, and branches (van Dixhoorn et al., 2016; Arroyo et al., 2020). However, how point-source environmental enrichment can modulate resilience in pigs is not known.

This study is part of a larger project which aims to identify predictors of resilience in young healthy pigs (Putz et al., 2019; Bai et al., 2020; Cheng et al., 2020). During the course of the project, phenotypes of resilience related traits were collected in more than 3,000 pigs after exposure to a natural polymicrobial challenge. Resilience traits measured included complete blood count (CBC), average daily gain (ADG), number of individual health treatments (nTRT), mortality, and feeding behavior (Putz et al., 2019; Bai et al., 2020; Cheng et al., 2020). More recently, the natural disease challenge model was also used to explore the impact of environmental enrichment on disease resilience, comparing a subset of approximately 400 pigs reared with and without point-source enrichment. Metabolite concentration was quantified in the plasma of 968 young healthy pigs, including some of those that received enrichment. The same metabolite data were previously analyzed to estimate heritability and genetic correlation between metabolites and production, resilience (e.g. number of treatments) and carcass traits (Dervishi et al., 2021b). Animals were classified into four groups (resilient, average, susceptible and dead) based on grow-to-finisher growth rate (GFGR) and treatment rate (TR). Furthermore, in a previous study using the same animals and group classification, resilient pigs were found to have greater increases of lymphocyte concentration after pathogen exposure and the magnitude of change in lymphocytes levels was reported to be heritable (0.11 ± 0.04; Bai et al., 2020). Although the magnitude of change in lymphocytes is attractive as a potential predictor trait for resilience, in practice it requires blood sampling before and after disease challenge, hence, more manual labor and animal manipulation. Therefore, the purpose of this study was to: 1) explore plasma metabolite levels in young healthy pigs and their potential association with health group classification after disease challenge, including the magnitude of change in lymphocytes levels following exposure to a polymicrobial disease challenge, 2) identify metabolic pathways that are impacted by point-source environmental enrichment or health group classification, and 3) explore the effect of the environmental conditions such as batch and entry age on metabolite concentrations.

## Materials and Methods

This study is part of a larger research project which attempts to elucidate the genetic mechanisms that underly disease resilience in nursery-grow-finisher pigs exposed to a natural polymicrobial disease challenge (Bai et al., 2020). The project also provided an opportunity to evaluate the impact of environmental enrichment on disease resilience using a subset of pigs. The Animal Care protocol was approved by the Animal Protection Committee of the Centre de Recherche en Sciences Animales de Deschambault (15PO283) and the Animal Care and Use Committee at the University of Alberta (AUP00002227). All animal handling was carried out in accordance with the Canadian Council on Animal Care guidelines (CCAC, 1993) and Animal Research: Reporting of In Vivo Experiments (ARRIVE) guidelines (https://arriveguidelines.org) (Percie du Sert et al., 2020).

### Animal, diets, and sample collection

The details of the natural polymicrobial challenge and phenotypes/traits that were collected including GFGR and TR were previously described in Putz et al. (2019), Cheng et al. (2020), and Bai et al. (2020). For purpose of clarity here, we briefly describe some of the details of the natural polymicrobial challenge.

Healthy F1 crossbred (Landrace × Yorkshire) castrated male weaned pigs were provided in rotation by seven genetic suppliers, all members of PigGen Canada. A total of 3205 pigs were introduced in 50 batches. All pigs were introduced into a natural disease challenge model. Each batch consisted of approximately 65 or 75 pigs from one of the genetic suppliers. After the pigs were weaned, they were transported to a healthy quarantine unit where they remained for a 3-wk period. After the quarantine nursery period, pigs were moved to a test station at the Centre de Développement du Porc du Québec (CDPQ), challenge nursery, where they remained for 4 wk and finally to a finisher barn where they remained for approximately 16 wk until fully recovered when they were sent for slaughter. The number of pigs per pen was approximately 4, 7, and 13 for the healthy quarantine unit, the challenge nursery, and the grow-to-finisher, respectively (Bai et al., 2020).

To establish the natural disease challenge model in 2015, naturally infected seeder animals with known diseases were introduced into the challenge nursery and finisher barn with the first four batches of healthy pigs, with the purpose of establishing a natural polymicrobial challenge within the unit (Bai et al. 2020; Cheng et al., 2020). Sustained pathogen transmission was established by introducing a new batch of healthy animals into the airspace containing the previous batch of pigs every 3 wk. The new and previous batches were maintained in separate pens, with pen-to-pen contact maintained for up to 1 wk in the challenge nursery to maintain a natural disease challenge. Through routine and reactive diagnostic testing, numerous respiratory, enteric and systemic pathogens have been identified in the natural disease challenge model including viruses (PRRSV and swine influenza virus A), bacterial pathogens (*Brachyspira hampsonii*, *Glaesserella parasuis*, *Mycoplasma hyopneumoniae*, *Salmonella enterica* serovar Typhimurium, and *Streptococcus suis*), and two parasites (*Ascaris suum* and *Cystoisospora suis*) (Bai et al., 2020). All pigs were fed the same commercial diet appropriate for the age and weight ad libitum (Délice, Nourisson and Premier Age (Cie Alfred Couture ltée; Quebec, Canada) in the quarantine nursery, challenge nursery, and finisher respectively).

Blood samples were collected from all pigs at four different time points. The first blood sample for was collected on all pigs in the quarantine nursery, 5 d postarrival from their farm of origin. The second blood sample was collected immediately before transferring to the challenge nursery. The third blood sample was collected on all pigs 2 wk after transferring to the challenge nursery. The fourth sample was collected 6 wk after the transfer to the challenge nursery. This study included plasma samples taken from 968 healthy pigs in 15 batches, (selected between batches 26 and 50), prior to challenge (first blood sample), which were used for metabolomics analysis.

All animals (*N* = 3,205) were genotyped using a 650k Affymetrix Axiom Porcine Genotyping Array by Delta Genomics (Edmonton AB, Canada). Raw Affymetrix SNP data were processed by Delta Genomics, separately for each cycle, with the Axiom Analysis Suite, using all defaults. Details of genotyping and quality control are described in Cheng et al. (2020) and Bai et al. (2020). After quality control, a total of 417,443 SNPs in 3205 pigs remained and were used for analysis.

### Environmental enrichment

**Figure 1.**
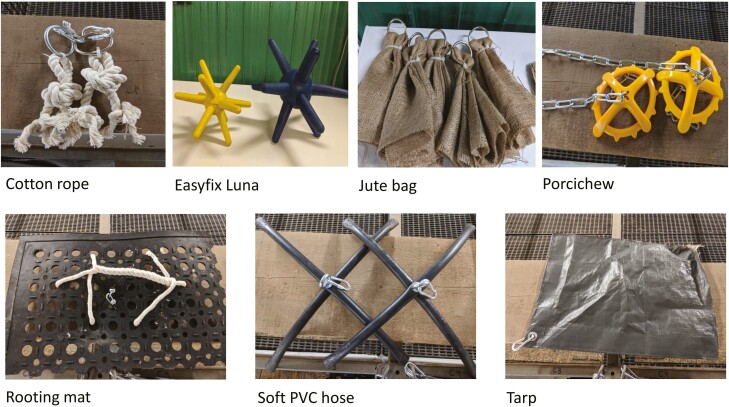
Enrichment environment objects provided to pigs.

Environmental enrichment (inedible point source objects) was provided in 50% of quarantine nursery pens in a subset of batches (*N* = 7 batches) with the purpose to evaluate the impact of environmental enrichment on disease resilience. In total, from pigs across all 15 batches, 763 of 968 (79%) pigs were raised in a barren environment and 205 (21%) pigs (from within 7 batches only) received enrichment. Chains were provided as a basic enrichment for barren pens in order to meet a minimal Code of Practice (NFACC, 2014) requirement. Enriched pens received a rotation of seven different suspended enrichment items consisting of a combination natural fibrous material and commercial pig enrichment containing the properties attractive to pigs (van de Weerd and Day, 2009): cotton rope, EasyFix Luna (EasyFix, Ballinasloe, Ireland), jute bag, porcichew (NutraPet, East Yorkshire, UK), a rooting mat (rubber boot mat with cotton rope threaded through for pigs to root and chew), soft PVC pipe, and tarpaulin (Figure 1). Each pen was provided with two of the same objects at one time, except for the EasyFix luna, in which one object per pen was given. Rotation of objects occurred three times a week (Monday, Wednesday, and Friday) in order to support novelty and retain interest (Trickett et al., 2009), and the order of presentation of objects was kept consistent for all batches. To prevent ingestion of cotton rope and jute sack, the enrichment was removed and a fresh piece added if the enrichment was starting to become tattered and close to breaking off, risking ingestion. Pigs that received point-source enrichment in the quarantine nursery, continued to receive enrichment in the challenge nursery and finisher. Mixing of pen groups occurred within treatment (as pen size changed across growth stages. The rooting mat and jute bag were not given to finisher pigs to prevent ingestion. The characteristics of environmental enrichment (inedible point source objects) are summarized in Supplementary Table S1.

### Classification of pigs

After slaughter, pigs within each batch were classified into four phenotypic groups: resilient (RES; *N* = 77), average (MID; *N* = 601), susceptible (SUS; *N* = 69), and dead (DEAD; *N* = 221). The classification was performed based on the GFRG and TR or mortality of each animal following the natural polymicrobial challenge. Within each batch slaughtered pigs that had equal or higher GFGR than the third quartile (Q3, 75% quartile) and equal or lower TR than the first quartile (Q1, 25% quartile) of all slaughtered pigs in the batch were classified as RES. Slaughtered pigs that had equal or lower GFGR than the Q1 and equal or higher TR than the Q3 of all slaughtered pigs in the batch were regarded as SUS. The rest of the slaughtered animals, with moderate TR and GFGR were classified as MID. Pigs that died as a result of infectious disease were classified as DEAD. Details of the classification are described by Bai et al. (2020). The number of pigs classified as dead, susceptible, average and resilient, by batch are shown in Supplementary Table S2.

### Nuclear magnetic resonance

Blood was collected in the quarantine nursery at an average age of 28 ± 3.23 d, 5 d postarrival from their farm of origin. In total, blood samples of 968 young healthy pigs introduced in 15 batches of 60 or 75 pigs were used for metabolomics analysis. Blood was collected from the jugular vein into K2 ethylenediaminetetraacetic acid (EDTA) tubes (BD Vacutainer Blood Collection Tubes, United States) and centrifuged at 3,000 rpm at 4 °C for 10 min. Plasma was collected and stored at −80 °C and only thawed for the metabolomics analysis.

Forty-eight plasma metabolites from eight analyte groups were quantified using NMR, following established protocols at The Metabolomics Innovation Center at University of Alberta (TMIC), AB, Canada (https://www.metabolomicscentre.ca/): amino acids, short chain fatty acids, sugars, alcohols, organic acids, amines, TCA cycle intermediates, and urea cycle intermediates. The details of the sample preparation and NMR protocol were described in details by Dervishi et al. (2021b). Briefly, after sample preparation, plasma samples (250 µL) were transferred in 3 mm SampleJet NMR tubes for spectral analysis. All 1H-NMR spectra were collected on a 700 MHz Avance III (Bruker) spectrometer equipped with a 5-mm HCN Z-gradient pulsed-field gradient (PFG) cryoprobe. 1H-NMR spectra were acquired at 25 °C using the first transient of the NOESY presaturation pulse sequence (noesy1dpr), chosen for its high degree of quantitative accuracy (Saude et al., 2006). All FID’s (free induction decays) were zero-filled to 250 K data points. The singlet produced by the DSS methyl groups was used as an internal standard for chemical shift referencing (set to 0 ppm) and for quantification all ^1^H NMR spectra were processed and analyzed using an in-house version of the MAGMET automated analysis software package using a custom metabolite library. MAGMET allows for qualitative and quantitative analysis of an NMR spectrum by automatically fitting spectral signatures from an internal database to the spectrum (Ravanbakhsh et al., 2015). An NMR spectroscopist inspected each spectrum in order to minimize compound misidentification and misquantification (Dervishi et al. 2021b).

### Statistical analysis

#### Plasma metabolite concentration

A quality control step was applied to the metabolite data prior to analysis and described by Dervishi et al. (2021b). Metabolites that were frequently (>20%) below the limit of detection or with at least 20% missing values were removed from consideration (acetic acid, isopropanol, ketoleucine, 3-hydroxyisovaleric acid). A total of 44 metabolites remained in the dataset. Data normalization (log10) of metabolite concentrations that were not normally distributed was done prior to statistical analysis. Three combinations of metabolites were also used for statistical analysis as described in Dervishi et al. (2021b): 1) branched chain amino acids (BCAA), which was calculated as the sum of L-leucine, L-isoleucine, and L-valine, 2) ketogenic amino acids (ketoAA), calculated as the sum of L-lysine and L-leucine, and 3) ketones, calculated as the sum of the three ketone bodies, 3-hydroxybutyric acid acetoacetate, and acetone.

Significance of group classification, batch, enrichment, and of the covariable age at entry was determined using the following linear mixed effects model implemented in R studio (2015) statistical software:


$$\begin{array}{l} Y=Group+Batch+NurEnrich+Age+Pen+Litter+e \end{array}$$

where *Υ* is the trait (metabolite concentration); Group is the group classification of pigs (resilient, middle, susceptible and dead); Batch is the fixed batch effect (*N* = 1 to 15); NurEnrich is the fixed effect of environmental enrichment (barren or enriched); Age is the covariate of age when the pig entered the quarantine nursery; Pen is the random effect of pen by batch in the quarantine nursery; Litter is the random effect of the litter environmental effect, and *e* is the error. The residuals of the model were plotted and visually inspected for the presence of outliers. To test differences between group classification and enrichment conditions, the Least Square Means (LSMs) for each pair-wise comparison were estimated and their *P* values were adjusted using false discovery ratio correction (FDR). Differences were considered significant if *P* ≤ 0.05 and as a tendency towards significance if *P* > 0.05 and ≤ 0.10.

#### Estimation of phenotypic and genetic correlations of metabolites with change of lymphocytes concentration following exposure

In a previous study using the same pigs, Bai et al. (2020) reported differences in complete blood count (CBC) between pigs classified as RES, MID, SUS, and DEAD. The most interesting result was that resilient pigs had a greater increase in lymphocyte count in blood collected in the quarantine nursery, at an average of 28 d, 5 d postarrival from their farm of origin (blood 1) and 2-wk after exposure to the natural disease challenge, at an average age of 54 d (blood 3) (LYMΔ13) when compared to the dead, susceptible, and middle groups (Supplementary Figure S1) (Bai et al., 2020). Therefore, we estimated the phenotypic and genetic correlations of the plasma concentrations in the quarantine nursery (blood 1) of 33 heritable metabolites (Dervishi et al., 2021b) with LYMΔ13 using bivariate models.

Phenotypic and genetic correlations were estimated by using the BLUPF90 programs (Misztal et al., 2002). The general model was as follows


$$\begin{array}{l} {Y_{ijk}}_{=}Batch_{i}+Age_{ijk}+Pen_{j}+Litter_{ijk}+u_{ijk}+e_{ijk} \end{array}$$

where *Y*_*ijk*_ is the trait (metabolite/LYMΔ13); Batch_*i*_ is the fixed batch effect (*i* = 1, …, 15); Age_*ijk*_ is the covariate of age when the pig entered the quarantine nursery; Pen_*j*_ is the random effect of pen by batch corresponding the quarantine nursery, with Pen_*j*_ ~ *N* (0, σ^2^_P_) where σ^2^_P_ is pen variance; *Litter*_*ijk*_ is the litter environmental effect, with Litter_*ijk*_ ~ *N* (0, σ^2^_L_) where σ^2^_L_ is the litter environmental variance; *u*_*ijk*_ is the random additive genetic effect, with the vector ***u*** ~ *N* (**0**, **G**σ^2^_A_), where **G** is the genomic relationship matrix and σ^2^_A_ is the additive genetic variance; and *e*_*ijk*_ is the residual effect, with *e*_*ijk*_ ~ N (0, σ^2^_e_) where σ^2^_e_ is the residual variance. The genomic relationship matrix, **G**, was created separately for each of the seven companies supplying pigs using the software preGSf90 (Misztal et al., 2002) and the method described by VanRaden (2008), and then combined into one **G** matrix, with genetic relationships between companies set to zero in order to focus on within-company variance components, as described by Cheng et al. (2020). For six metabolites namely: 3-hydroxybutyric acid, 3-methyl-2-oxovaleric acid, formate, L-asparagine, L-leucine and L-serine, environment enrichment was included as a fixed effect because the effect of enrichment was significant (*P* ≤ 0.05). The model used for LYMΔ13 was the same as for metabolites, except pen was not included as random effect because it was not significant as described by Bai et al. (2020). For estimation of phenotypic and genetic correlations, the group classification was not included in the model because the group classification was carried out after slaughter.

Genetic correlations between two traits were estimated as the estimate of the genetic covariance from the bivariate analysis divided by the product of the genetic standard deviations for the two traits. A likelihood ratio test (LRT) test was conducted to establish the significance of genetic and phenotypic correlations. Correlations were considered significant when *P* ≤ 0.05.

#### Functional annotation analyses

Metabolite set enrichment analysis (MSEA) was carried out using www.metaboanalyst.ca (Xia et al., 2016). Significant metabolites (*P* ≤ 0.05) and those that tended to be significantly (*P* > 0.05 and ≤ 0.10) affected either by group or the environmental enrichment were considered for enrichment analysis separately. This analysis identifies which metabolic pathways have compounds (from the input lists) that are overrepresented. For enrichment analysis, we used hypergeometric and Fisher’s exact tests of significance.

## Results

Concentration of all metabolites was affected by batch, while entry age was the second most important factor affecting the concentration of 16 metabolites, BCAA index, and ketone bodies, followed by environmental enrichment and group classification (Table 1). Overall, creatinine and hypoxanthine had the lowest coefficient of variation (cv) by batch. The minimum, maximum, and cv of 33 heritable metabolites by batch it is shown in Supplementary Table S3.

**Table 1.** Significance of fixed effects (*F*-value and Pr(>F)) using type III analysis of variance with Satterthwaite’s method (*N* = 968 pigs)

Metabolite μM	Batch^2^	Group	Nursery enrichment	Entry age
1-Methylhistidine	4.52 (<0.0001)^***^	0.42 (0.73)	1.41 (0.23)	5.02 (0.02)^*^
2-hydroxybutyrate log_10_^1^	12.35 (<0.0001)^***^	1.94 (0.12)	0.22 (0.63)	5.91 (0.01)^*^
2-hydroxyisovalerate log_10_	5.88 (<0.0001)^***^	0.34 (0.79)	0.07 (0.79)	0.36 (0.54)
3-Hydroxybutyric acid log_10_	7.74 (<0.0001)^***^	0.05 (0.98)	5.68 (0.01)^*^	2.43 (0.11)
3-Methyl-2-oxovaleric acid	15.23 (<0.0001)^***^	2.44 (0.06) ^x^	4.49 (0.03)^*^	2.78 (0.09)^x^
Acetoacetate log_10_	7.31 (<0.0001)^***^	2.70 (0.04)^*^	1.09 (0.29)	5.15 (0.02)^*^
Acetone log_10_	19.60 <0.0001)^***^	0.98 (0.39)	0.458 (0.49)	0.62 (0.43)
Betaine	8.34 (<0.0001)^***^	1.36 (0.25)	0.005 (0.94)	2.65 (0.10)^x^
Citric acid	6.77 (<0.0001)^***^	0.77 (0.50)	0.24 (0.62)	0.94 (0.33)
Creatine log_10_	8.16 (<0.0001)^***^	1.03 (0.37)	0.51 (0.47)	2.10 (0.14)
Creatinine	7.29 (<0.0001)^***^	3.84 (0.009)^**^	2.05 (0.15)	0.34 (0.55)
Dimethylglycine log_10_	21.90 (<0.0001)^***^	1.57 (0.19)	1.25 (0.26)	0.04 (0.83)
Ethanol log_10_	11.62 (<0.0001)^***^	0.43 (0.73)	0.08 (0.77)	2.95 (0.08)^x^
Formate log_10_	92.49 (<0.0001)^***^	1.00 (0.38)	4.07 (0.04)^*^	0.02 (0.87)
D-glucose	26.75 (<0.0001)^***^	0.49 (0.68)	1.24 (0.26)	3.72 (0.05)^*^
Glycerol	16.55 (<0.0001)^***^	0.34 (0.79)	1.02 (0.31)	0.64 (0.42)
Hypoxanthine	32.98 (<0.0001)^***^	0.74 (0.52)	0.03 (0.85)	8.48 (0.003)^**^
Isobutyric acid	8.34 (<0.0001)^***^	0.15 (0.92)	0.001 (0.97)	12.63 (0.0004)^***^
L-acetylcarnitine	8.93 (<0.0001)^***^	0.11 (0.95)	1.06 (0.30)	1.95 (0.16)
L-alanine	10.30 (<0.0001)^***^	0.31 (0.81)	1.82 (0.17)	6.84 (0.009)^**^
L-arginine	2.72 (0.001)^**^	0.21 (0.88)	0.16 (0.68)	0.31 (0.57)
L-aspartate	8.85 (<0.0001)^***^	0.34 (0.79)	0.05 (0.82)	0.19 (0.65)
L-asparagine	12.09 (<0.0001)^***^	1.76 (0.15)	9.35 (0.002)^**^	1.09 (0.29)
L-glutamine	7.84 (<0.0001)^***^	0.40 (0.74)	2.04 (0.15)	0.32 (0.56)
L-glutamic acid	11.03 (<0.0001)^***^	0.04 (0.98)	1.44 (0.23)	4.25 (0.04)^*^
L-glycine	9.57 (<0.0001)^***^	1.24 (0.29)	0.39 (0.52)	1.51 (0.22)
L-histidine	5.27 (<0.0001)^***^	0.48 (0.69)	0.70 (0.40)	0.09 (0.75)
L-isoleucine log_10_	19.68 (<0.0001)^***^	2.96 (0.03)^*^	2.39 (0.12)	0.003 (0.95)
L-leucine	6.79 (<0.0001)^***^	2.22 (0.08) ^x^	5.06 (0.02)^*^	3.31 (0.06)^x^
L-lysine	13.25 (<0.0001)^***^	1.28 (0.28)	2.14 (0.14)	0.07 (0.77)
L-methionine	10.50 (<0.0001)^***^	0.45 (0.71)	1.26 (0.26)	0.80 (0.37)
L-proline	21.40 (<0.0001)^***^	0.58 (0.62)	2.35 (0.12)	0.50 (0.47)
L-phenylalanine	4.58 (<0.0001)^***^	1.70 (0.16)	1.22 (0.26)	3.48 (0.06)^x^
L-serine	15.36 (<0.0001)^***^	1.45 (0.22)	4.07 (0.04)^*^	0.41 (0.52)
L-ornithine	22.29 (<0.0001)^***^	0.64 (0.58)	3.18 (0.07) ^x^	11.20 (<0.001)^***^
L-threonine	15.82 (<0.0001) ^***^	2.47 (0.06) ^x^	0.16 (0.68)	5.82 (0.02)^*^
L-tyrosine	8.28 (<0.0001)^***^	0.31 (0.81)	1.86 (0.17)	9.22 (0.002)^**^
L-valine	10.96 (<0.0001)^***^	0.71 (0.54)	3.29 (0.07)^x^	9.06 (0.002)^**^
L-alpha-aminobutyric acid log_10_	18.87 (<0.0001)^***^	0.90 (0.43)	0.008 (0.92)	8.00 (0.004)^**^
L-lactic acid	9.93 (<0.0001)^***^	1.17 (0.31)	0.00 (0.99)	0.01 (0.90)
Methanol log_10_	10.10 (<0.0001)^***^	1.84 (0.13)	0.55 (0.45)	4.19 (0.04)^*^
Oxoglutarate	13.26 (<0.0001)^***^	0.62 (0.59)	0.59 (0.44)	5.23 (0.02)^*^
Pyruvic acid	23.37 (<0.0001)^***^	0.75 (0.51)	0.15 (0.69)	4.46 (0.03)^*^
Succinate	14.97 (<0.0001)^***^	0.86 (0.45)	0.56 (0.45)	0.28 (0.59)
BCAA	11.67 (<0.0001)^***^	1.12 (0.33)	5.01 (0.02)^*^	5.58 (0.01)^*^
KetoAA	9.66 (<0.0001)^***^	1.44 (0.22)	3.42 (0.06)^x^	0.58 (0.44)
Ketone bodies log_10_	10.55 (<0.0001)^***^	0.79 (0.49)	0.44 (0.50)	5.15 (0.02)^*^

^1^Log_10_: metabolite normalization; ^2^Significance of fixed effects are indicated as: ^***,**,*,x^ corresponding to *P* < 0.001, *P* < 0.01, *P* < 0.05, and 0.05 < *P* < 0.10, respectively.

### The effect of phenotypic group classification on the concentration of metabolites

The results comparing the least-squares means of metabolites in groups with different responses to the natural disease challenge are shown in Table 2. Creatinine was significantly lower for pigs that died before marketing, classified as “dead” and “SUS” when compared to “MID” group (*P* < 0.05). In addition, group classification tended to be significant for acetoacetate, isoleucine, 3-methyl-2-oxovaleric acid, L-leucine, and L-threonine. Pigs classified as MID and RES tended to have higher acetoacetate and L-threonine concentration when compared with pigs that died before harvest (*P* > 0.05 and ≤ 0.10). MID pigs tended to have lower concentration of 3-methyl-2-oxovaleric acid when compared to the dead group. Finally, RES pigs tended to have lower concentration of L-leucine when compared to SUS and MID pigs (*P* > 0.05 and ≤ 0.10).

**Table 2.** Least-squares means and standard errors (SE) of significant metabolites in pigs with different responses to the natural polymicrobial challenge: dead, susceptible, average, and resilient

Metabolites μM	DEAD (*N* = 221)	SUS (*N* = 69)	MID (*N* = 601)	RES (*N* = 77)	FDR^2^
Creatinine	81.1 (0.98)^a^	79.8 (1.58)^a^	83.7 (0.75)^b^	82.8 (1.51)^a,b^	0.03
Acetoacetate log_10_^1^	1.29 (0.03)^x^	1.33 (0.05)^x,y^	1.36 (0.02)^y^	1.42 (0.04)^y^	0.06
Isoleucine log_10_	3.72 (0.03)^x,y^	3.78 (0.05)^x^	3.64 (0.02)^y^	3.61 (0.05)^y^	0.1
L-Leucine	106 (2.00)^x,y^	110 (3.27)^x^	108 (1.49)^x^	101 (3.09)^y^	0.06
L-Threonine	377 (13.50)^x^	417 (21.90)^x,y^	409 (10.20)^y^	425 (20.80)^y^	0.1
3-Methyl-2-oxovaleric acid	4.83 (0.17)^x^	4.81 (0.27)^x,y^	4.40 (0.13)^y^	4.48 (0.26)^x,y^	0.08

^1^Log_10_: metabolite normalization; ^2,a,b,c^: within a row least square mean with different subscript differ at *P <* 0.05 after FDR correction and ^x,y^: within a row least square mean with different subscript tended to differ at *P* > 0.05 and ≤ 0.10 after FDR correction.

**Figure 2.**
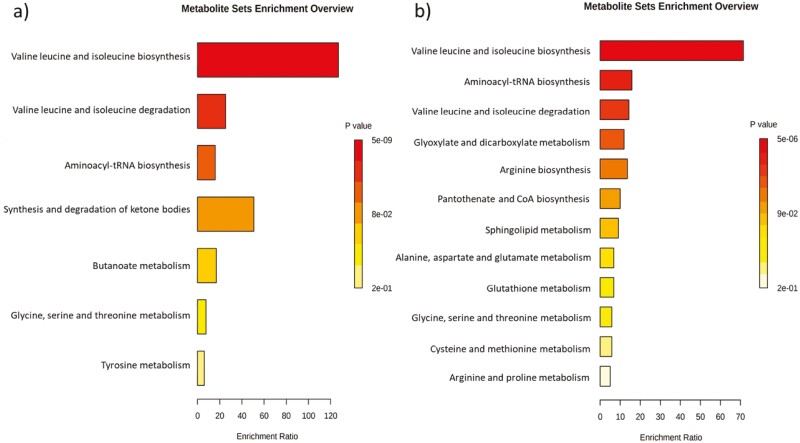
a) Pathways that are significantly enriched when using group classification of pigs with different responses to the natural polymicrobial challenge and b) pathways that are significantly enriched when using environmental enrichment. Enrichment Ratio is computed by Hits / Expected, where hits = observed hits; expected = expected hits.

Pathway enrichment analysis (Figure 2a) showed that these metabolites are involved in the valine, leucine, and isoleucine biosynthesis (FDR = 3.94e−7) and degradation pathways (FDR = 0.0002).

### The effect of environmental enrichment on the concentration of metabolites

Providing enrichment significantly affected the concentration of six metabolites and BCAA index (*P* ≤ 0.05). Pigs that received enrichment during the experiment had lower concentration of 3-hydroxybutyric acid, 3-methyl-2-oxovaleric acid, formate, L-asparagine, L-leucine, and L-serine when compared with pigs that did not receive enrichment (Table 3). Moreover, enrichment tended to affect the concentration of the amino acids L-ornithine, L-valine, and the index of ketoAA (*P* > 0.05 and ≤ 0.10). Pathway enrichment analysis (Figure 2b) showed that these metabolites are involved in the valine, leucine, and isoleucine biosynthesis (FDR = 0.0004) and degradation pathways (FDR = 0.024).

**Table 3.** Least-squares means ± standard errors for metabolites of animals from barren and enriched environment

Metabolite μM	Barren (*N* = 763)	Enriched (*N* = 205)	FDR^2^
3-Hydroxybutyric acid log_10_^1^	0.48 (0.01)	0.44 (0.01)	0.01
3-Methyl-2-oxovaleric acid	4.89 (0.12)	4.37 (0.22)	0.03
BCAA	361 (4.43)	342 (7.63)	0.02
Formate log_10_	2.07 (0.01)	2.01 (0.02)	0.05
L-Asparagine	50.2 (0.84)	45.4 (1.44)	0.004
L-Leucine	109 (1.47)	103 (2.53)	0.04
L-Serine	188 (2.40)	179 (3.89)	0.04
L-Valine	203 (2.51)	193 (4.36)	0.07
L-Ornithine	95.7 (2.08)	89.1 (3.45)	0.07
ketoAA	372 (4.90)	355 (8.22)	0.06

^1^Log_10_ metabolite normalization.

^2^ *P-*value after FDR correction.

### Genetic and phenotypic correlations between metabolites and magnitude of change of lymphocytes

**Figure 3.**
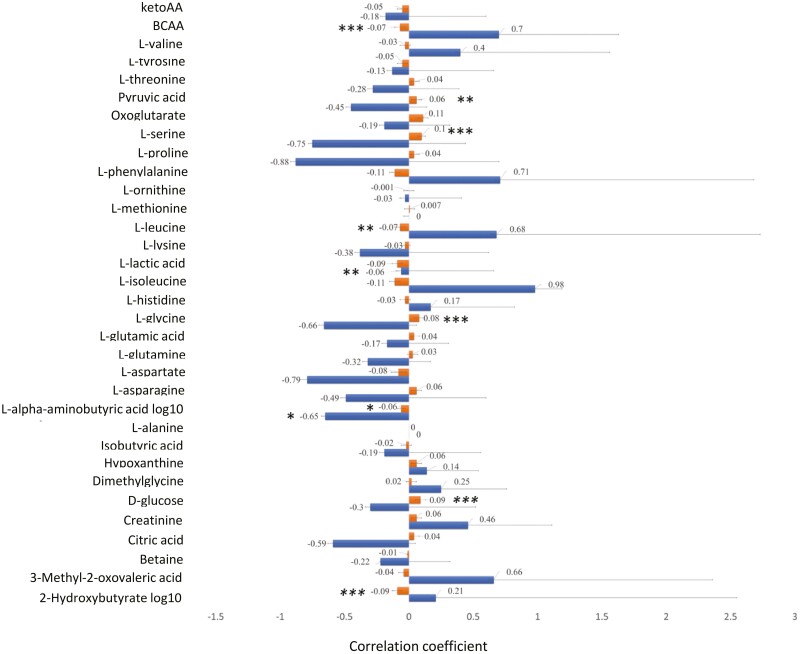
Estimates of genetic (blue color) and phenotypic correlations (orange color) between thirty-three heritable metabolites and LYMΔ13. Significance of correlations are indicated as: ***, **, * corresponding to P < 0.001, P < 0.01, and P < 0.05 respectively.

Overall genetic correlation between metabolites and LYMΔ_13_ were low with high standard errors (SE) (Figure 3). Only one metabolite, L-α-aminobutyric acid, was negatively genetically correlated with LYMΔ_13_ (−0.65 ± 0.51; *P* < 0.05). There was a tendency for L-aspartate to be genetically negatively correlated with LYMΔ_13._

Phenotypic correlation between metabolites and LYMΔ_13_ were very low. However, a few metabolites (L-glycine, oxoglutarate, D-glucose, pyruvic acid, and L-serine) were significantly positively correlated with LYMΔ_13_ (Figure 3; *P* < 0.05). L-aspartate, L-alpha- aminobutyric acid and BCAA were phenotypically negatively correlated with LYMΔ_13._

## Discussion

In this study, we explored plasma metabolite concentrations in young healthy pigs and their association with disease resilience in pigs with different responses to the natural polymicrobial challenge. The metabolomics samples were collected from 968 healthy pigs at an average of 28 d of age in the quarantine nursery. The final goal of the project was to elucidate the genetic foundation of disease resilience in pigs in order to select resilient pigs to improve their production, health, and welfare. In addition, this project attempts to explore the impact of environmental enrichment on disease resilience. Such results would be very useful to design environmental enrichment strategies which would enhance resilience and provide a better health outcome when pigs are challenged by disease in commercial farms.

In the present study, all metabolites were affected by batch in agreement with a previous study reported by Dervishi et al. (2021a). Variation in metabolite concentration between batches can be due to a number of factors such as weaning stress, transportation, and genetics. In this study, all pigs were provided in rotation by seven genetic suppliers, members of PigGen Canada. Before entering the experiment, pigs were weaned and transported from their farm of origin to Centre de Développement du Porc du Québec (CDPQ) and transportation time was different for each batch of pigs. It has been reported that weaning stress can result in imprairment of immune status, hepatic dysfunction, and hepatic metabolites, therefore hindering the pig’s health and growth performance (Southey et al., 2021). In addition, each batch of pigs was provided by one of the seven genetic suppliers, suggesting that a genetic effect may also be associated with batch. Another factor that is accounted for by “batch” is the season of the year. Therefore, a possible effect of genetics, weaning stress, transportation, and season on metabolite concentration cannot be excluded.

The second most important factor that affected metabolite concentration was the entry age (16 metabolites). Pigs were weaned and entered the experiment at different ages (average 28 d (SD 3.23). It was expected that entry age will affect the concentration of metabolites as pigs undergo major physiological changes at weaning time. Previous studies have shown an effect of age on cellular and enzymatic activity in porcine intramuscular adipose tissue (Lee and Kauffman, 1974; Etherton et al., 1981) therefore, it is expected that the concentration of metabolites that are the result of these enzymatic reactions would also be affected by age. Yu et al. (2012) found strong associations between age and the human metabolome. In addition, (Vignoli et al. (2018) reported an effect of age on metabolite concentration in human. Furthermore, in pigs, using samples collected on weeks 8, 9, and 22 of life, Dervishi et al. (2021a) showed an effect of age of the pigs on metabolite concentration. In the present study, the differences in entry age between pigs are small. However, even very small differences in age can have an impact on metabolites concentration and should be taken into account.

In the current study, small significant differences in plasma creatinine between the classification groups was found. Creatinine concentrations of pigs that died in the challenge stages and in SUS pigs was lower than in MID pigs. Creatinine, a waste product generated from muscle metabolism, is cleared from the body by the kidney. When not removed effectively by the kidneys, creatinine levels will rise in blood. It has been proposed that creatinine levels in blood can be used as an indicator of protein deposition (Cameron et al., 2003) and as an indicator of muscle proteolysis in pigs challenged by heat stress and PRRS virus (Seelenbinder et al., 2018). However, in our study, blood samples were collected when pigs were still healthy and the resilience classification was done based on GFRG and number of treatments after the natural polymicrobial challenge. Our results suggest that DEAD pigs and SUS pigs had lower muscle deposition therefore lower creatinine concentration when they were healthy compared to MID pigs. It has been reported in human medicine that low serum creatinine levels and muscle mass predict mortality in critically ill patients (Thongprayoon et al., 2016). In a recent study using the same data, we found that creatinine concentration was genetically correlated with mortality in the challenge nursery (Dervishi et al., 2021b). In addition, creatinine was genetically and phenotipically negatively correlated with ADG in quarantine nursery (Dervishi et al., 2021b) suggesting low ADG pigs, have high creatinine concentration. Animals with low ADG are expected to have lower protein or muscle deposition. It is possible that young healthy nursery pigs that have low ADG, and high plasma creatinine, are more likely to die when challenged by disease. Further studies are necessary to confirm whether creatinine concentration can be used as an early indicator of death and/or susceptibility of disease in pigs.

Resilient pigs tended to have higher concentration of L-threonine and acetoacetate when compared with animals that died before harvest. Threonine has been associated with protein synthesis in the gut (Schaart et al., 2005) and maintenance of gut barrier integrity (Yang and Liao, 2019). A decrease in threonine has been associated with ileal villous hypotrophy (Hamard et al., 2007) and decreased growth of weaned piglets. It is possible that resilient animals have a healthier and better developed gut structures at a young age compared with those in the other groups. Here, we show that threonine concentration tended to be higher in pigs classified as resilient later in life, therefore a relationship between threonine and resilience outcome later in life is not excluded.

We further explored the effect of environmental enrichment on metabolite concentration in the challenge model. Previous studies have shown that enrichment decreases levels of cortisol and lactate in the plasma of pigs (Arroyo et al., 2020) and that enrichment modulates monoaminergic neurotransmitter level in several brain areas and protein synthesis in the hippocampus (Arroyo et al., 2020). In our study, enrichment affected the concentration of six metabolites (3-hydroxybutyric acid, 3-methyl-2-oxovaleric acid, formate, L-asparagine, L-leucine, L-serine) and the index of BCAA. 3-hydroxybutyric acid (BHBA) is a ketone body, an intermediate metabolite of nonesterified fatty acids (NEFA) catabolism and the ketogenic amino acids, lysine and leucine, are metabolized to BHBA. During states of energy deficit, ketone bodies are produced in the liver and serve as an alternative source of energy for the brain, heart, and skeletal muscle, however, in high concentrations they cause damage. High concentration of BHBA is widely accepted as a diagnostic marker of ketosis in dairy cows (Ospina et al., 2010), however, in swine, this condition is not reported in the literature. In our study, we found that pigs that received enrichment had lower concentrations of leucine, ketoAA, and BHBA in serum suggesting lower mobilization of NEFA and probably better energy status compared with pigs that were housed in a barren environment. Interestingly in a previous study, we reported enriched housed pigs (straw bedding) had lower 3-methyl-2-oxovaleric acid concentration in plasma when compared with those in the barren environment (Dervishi et al., 2021a). 3-methyl-2-oxovaleric acid is a keto-acid and arises from the incomplete breakdown of branched-chain amino acids (Przyrembel et al., 1979). Branched amino acids are very important in mounting an immune response as they are essential for lymphocyte responsiveness and are necessary to support other immune cell functions (Calder, 2006). Here, we also found that circulating plasma BCAA levels were lower in pigs housed in an enriched environment, which suggests that these pigs might have a better utilization of amino acids and/or less break down of muscle tissue. In addition, MID pigs tended to have lower concentration of 3-methyl-2-oxovaleric acid when compared to DEAD pigs.

Interestingly, metabolite enrichment analysis showed that metabolites affected by group classification or enrichment are involved in the same metabolic pathways: valine, leucine, and isoleucine biosynthesis and degradation pathways. This result might indicate a connection between enrichment and health outcome. It is possible that providing enrichment might modulate health outcome in pigs. Further research is necessary to validate our findings. There is evidence linking environmental enrichment to positive effects on behavioral state, immunological response, and clinical outcome in pigs infected with PRRSV and *Actinobacillus pleuropneumoniae* (van Dixhoorn et al., 2016). It should be noted that the piglets had only been exposed to enrichment for 5 to 7 d at the time the blood sample was collected. At this early exposure, the enrichment is highly novel to the pigs and elicits a higher level of interactions. This is a short time of exposure, however, it was enough to significantly affect the concentration of metabolites which demonstrates the potential value of providing point-source enrichment to pigs and retain novelty.

In the present study, we estimated genotypic and phenotypic correlations between metabolites and LYMΔ_13._ The reason for selecting LYMΔ_13_ was because resilient pigs were found to have greater increase of lymphocyte concentration after pathogen exposure when compared with other groups (Bai et al., 2020). Although LYMΔ_13_ is attractive as a potential predictor trait for resilience, in practice it requires blood sampling before and after disease challenge, hence, more manual labor and animal manipulation. Therefore, an early indicator of LYMΔ_13_ that can be collected from nucleus breeding herds in healthy animals could provide a biomarker of resilience and would avoid collecting blood twice and reduce animal handling. Although relatively large SE were found, highly negative genetic correlations between L-alpha aminobutyric acid with LYMΔ_13_ (−0.65 ± 0.51) are reported here. In addition, the phenotypic correlation of L-α aminobutyric acid with LYMΔ_13_ was negative. α-Aminobutyric acid together with the neurotransmitter γ-aminobutyric acid (GABA) and β-aminobutyric acid (BABA) are isomers of aminobutyric acid. β-Aminobutyric acid, has been associated with plant disease resistance and affects the availability and the distribution of iron (Zimmerli et al., 2001; Koen et al., 2014). α-Aminobutyric (homoalanine) acid it is primarily derived from the catabolism of methionine, threonine, and serine (Newsholme and Leech, 1983; Darling et al., 2000). Also, it is formed by transamination of α-ketobutyric acid, a metabolite in BCAA biosynthesis (Yudkoff et al., 1979). Elevated levels of L-α aminobutyric acid in the plasma of children have been associated with Reye’s syndrome, tyrosinemia, homocystinuria, nonketotic hyperglycinemia, and ornithine transcarbamylase deficiency (Haschke-Becher et al., 2016). In addition, in humans, it has been suggested as a marker of the severity of sepsis (Chiarla et al., 2011). Interestingly in swine, increased levels of α-aminobutyric acid have been associated with presence of *Mycoplasma hyopneumoniae*, a pathogenic bacterium causing bronhopneumonia (Surendran Nair et al., 2019). It is worth mentioning that this bacterium was also shown to be present in the natural disease challenge model used here. The role of L-αaminobutyric acid in porcine physiology and disease is presently unknown, and its significance in the context of resilience has yet to be clarified. However, we speculate that L-α aminobutyric acid modulates the proliferation of lymphocytes after infection. Lymphocytes were reported to be related to lower TR and an increase in lymphocytes after pathogen exposure favors resilience in pigs (Bai et al., 2020). The genetic and phenotypic correlations reported here indicate that higher L-α aminobutyric acid at the quarantine nursery stage might delay an increase in lymphocytes after infection therefore negatively impacting resilience. To our best knowledge, this is the first study to report genetic correlations between metabolites and the magnitude of change in lymphocyte concentration in pigs.

## Conclusions

Pigs classified as susceptible and/or dead after disease challenge pigs had lower creatinine concentration as healthy nursery pigs. L-α-aminobutyric may be a genetic candiate biomarker for the magnitude of lymphocyte response after pathogen exposure and deserves further validation. Future studies are necessary to investigate the role of L-α-aminobutyric on lymphocyte proliferation following disease challenge. Providing environmental enrichment affects metabolites which are involved in the valine, leucine, and isoleucine biosynthesis and degradation pathways. Long term studies providing point-source enrichment are necessary to understand the role of environmental enrichment on disease resilience. Finally, batch and entry age, were important factors affecting the concentration of metabolites and should be taken into consideration for interpretation of results.

## Supplementary Material

skad033_suppl_Supplementary_Figure_S1

skad033_suppl_Supplementary_Table_S1

skad033_suppl_Supplementary_Table_S2

skad033_suppl_Supplementary_Table_S3

## Abbreviations

ADG: average daily gain
BCAA: branched chain amino acids
CBC: complete blood count
CDPQ: test station at the Centre de Développement du Porc du Québec
FDR: false discovery ratio correction
FID: free induction decay
GFGR: growth rate in the grow-to-finish phase
ketoAA: ketogenic amino acids
LRT: likelihood ratio test
LSM: least Square Means
LYMΔ13: magnitude of change in lymphocyte concentration
MID: average
MSEA: metabolite set enrichment analysis
NMR: Nuclear magnetic resonance
PFG: pulsed-field gradient
PRRSV: porcine reproductive and respiratory syndrome virus
RES: resilient
SUS: susceptible
TR: treatment rate
