# Supplementary material for: Exploration of plasma metabolite levels in healthy nursery pigs in response to environmental enrichment and disease resilience
Source: J Anim Sci. 2023 Jan 27;101:skad033. doi: 10.1093/jas/skad033 (PMC9982359; doi:10.1093/jas/skad033)
Supplement: skad033_suppl_Supplementary_Figure_S1 [file skad033_suppl_supplementary_figure_s1.docx]

**Supplementary Figure S1.** The increase in lymphocyte between blood 1 and blood 3 (LYMΔ_13_) in animals classified as resilient, average, dead, and susceptible (adopted by Bai et al., 2020).
