# Supplementary material for: Exploration of plasma metabolite levels in healthy nursery pigs in response to environmental enrichment and disease resilience
Source: J Anim Sci. 2023 Jan 27;101:skad033. doi: 10.1093/jas/skad033 (PMC9982359; doi:10.1093/jas/skad033)
Supplement: skad033_suppl_Supplementary_Table_S1 [file skad033_suppl_supplementary_table_s1.docx]

**Supplementary Table S1**. Characteristics of enrichment objects.

| **Enrichment** | **Details** | **Sizes Used** | **Attractive properties** |
| --- | --- | --- | --- |
|  |  |  |  |
| Porcichew  (NutraPet, East Yorkshire, UK) | Vanilla scented and flavored, anti-bacterial properties  Suspended from the ceiling by a carabiner clip and chain | One size | Odorous  Malleable |
|  |  |  |  |
| EasyFix Luna (EasyFix, Ballinasloe, Ireland) | Food grade rubber ball with 12 protruding spikes  Placed on the floor | Quarantine nursery- Luna 50  Challenge nursery - Luna 86  Finisher- Luna 117 | Manipulable  Malleable |
|  |  |  |  |
| Twisted cotton rope | Length of cotton rope tied in a knot to make two strands  Replaced between batches and if it became worn | Quarantine- ~12" in length, 1/2" diameter  Challenge nursery- ~18" in length, 5/8" diameter  Finisher- ~24" in length, 5/8" diameter | Malleable  Deformable |
|  |  |  |  |
| Soft PVC pipe | Two lengths of pipe crossed in an "X", attached by nut and bolt  Suspended from the ceiling  Replaced between batches | Quarantine- two tubes ~18" each, 1/2" diameter  Challenge nursery- two tubes ~24" each, 1/2" diameter  Finisher- two tubes ~36" each, 3/4" diameter | Malleable |
|  |  |  |  |
| Tarpaulin | Square of tarpaulin  Suspended from the ceiling  Replaced between batches and if it became worn | Quarantine- ~12" square  Challenge nursery- ~18" square  Finisher- ~24" square | Deformable |
|  |  |  |  |
| Jute sack | Rectangle of jute sack, folded lengthwise over a metal ring, gathered with a plastic cable tie  Suspended from the ceiling  Replaced if it became worn and between batches | Quarantine, challenge nursery - finished size ~10" long x 6" wide | Deformable |
|  |  |  |  |
| Rooting mat | Square of rubber anti-fatigue mat with cotton rope woven through the mat  Suspended from the ceiling  Washed between batches and reused, rope replaced when worn | Quarantine and challenge nursery: 12" square + ~12” of rope interwoven into the mat. | Malleable |
|  |  |  |  |
