# Supplementary material for: Exploration of plasma metabolite levels in healthy nursery pigs in response to environmental enrichment and disease resilience
Source: J Anim Sci. 2023 Jan 27;101:skad033. doi: 10.1093/jas/skad033 (PMC9982359; doi:10.1093/jas/skad033)
Supplement: skad033_suppl_Supplementary_Table_S2 [file skad033_suppl_supplementary_table_s2.docx]

**Supplementary Table S2.** The number of animals by batch classified into four groups with different responses to the natural polymicrobial challenge: dead, susceptible, average and resilient.

| **Batch** | | | | | | | | | | | | | | | |
| --- | --- | --- | --- | --- | --- | --- | --- | --- | --- | --- | --- | --- | --- | --- | --- |
|  | **1** | **2** | **3** | **4** | **5** | **6** | **7** | **8** | **9** | **10** | **11** | **12** | **13** | **14** | **15** |
| **Group** |  |  |  |  |  |  |  |  |  |  |  |  |  |  |  |
| **Dead** | 9 | 5 | 12 | 13 | 13 | 13 | 34 | 0 | 14 | 19 | 18 | 22 | 26 | 12 | 11 |
| **SUS** | 5 | 3 | 5 | 6 | 5 | 6 | 4 | 0 | 6 | 6 | 3 | 4 | 5 | 5 | 6 |
| **MID** | 55 | 49 | 39 | 51 | 37 | 47 | 21 | 62 | 33 | 29 | 30 | 40 | 22 | 42 | 44 |
| **RES** | 6 | 3 | 4 | 4 | 5 | 4 | 0 | 0 | 8 | 5 | 6 | 4 | 5 | 9 | 14 |
