# Supplementary material for: Exploration of plasma metabolite levels in healthy nursery pigs in response to environmental enrichment and disease resilience
Source: J Anim Sci. 2023 Jan 27;101:skad033. doi: 10.1093/jas/skad033 (PMC9982359; doi:10.1093/jas/skad033)
Supplement: skad033_suppl_Supplementary_Table_S3 [file skad033_suppl_supplementary_table_s3.docx]

Supplementary Table S3. The minimum (min), maximum (max) and coefficient of variation (cv) of 33 heritable metabolites in batch 1-8.

| **Batch** | **1** | | **2** | | **3** | | **4** | | **5** | | **6** | | **7** | | **8** | |
| --- | --- | --- | --- | --- | --- | --- | --- | --- | --- | --- | --- | --- | --- | --- | --- | --- |
| **Metabolite μM** | **min-max** | **CV** | **min-max** | **CV** | **min-max** | **CV** | **min-max** | **CV** | **min-max** | **CV** | **min-max** | **CV** | **min-max** | **CV** | **min-max** | **CV** |
| 2-Hydroxybutyrate | 8.86- 168.55 | 0.71 | 7.52- 233.66 | 1.21 | 9.71- 58.41 | 0.42 | 7.42- 66.7 | 0.54 | 9.23- 546.44 | 2.24 | 8.97- 96.55 | 0.71 | 10.67- 75.44 | 0.54 | 6.16- 35.48 | 0.38 |
| 3-Methyl-2-oxovaleric acid | 3.92- 14.89 | 0.33 | 2.42- 14.85 | 0.41 | 2.36- 14.87 | 0.47 | 1.98- 11.5 | 0.45 | 1.72- 11.65 | 0.47 | 1.93 -12.45 | 0.42 | 1.7- 11.58 | 0.48 | 2.06- 8.65 | 0.36 |
| Betaine | 8.66- 126.24 | 0.51 | 35.13- 155.29 | 0.34 | 23.95- 188.19 | 0.43 | 15.32- 171.66 | 0.43 | 25.52- 154.65 | 0.41 | 22.3- 142.23 | 0.33 | 24.27- 132.1 | 0.37 | 44.18- 165.7 | 0.32 |
| Citric acid | 67.87- 358.57 | 0.34 | 32.4- 316.28 | 0.27 | 70.42- 319.69 | 0.21 | 34.28 -294.69 | 0.21 | 53.6- 289.25 | 0.29 | 118.27- 289.23 | 0.20 | 65.06- 380.5 | 0.24 | 110.3- 379.3 | 0.23 |
| Creatinine | 54.1- 124.32 | 0.17 | 55.54- 103.9 | 0.13 | 48.79-112.09 | 0.16 | 55.59- 116.76 | 0.14 | 65.8- 138.39 | 0.19 | 58.27- 108.16 | 0.13 | 50.13- 108.0 | 0.16 | 51.42- 97.75 | 0.12 |
| D-glucose | 179-4623 | 0.36 | 88-3956 | 0.48 | 1261-5018 | 0.31 | 939-5476 | 0.25 | 43-4023 | 0.50 | 49-4199 | 0.44 | 30-5151 | 0.43 | 2975-5126 | 0.14 |
| Dimethylglycine | 1.3- 11.34 | 0.62 | 1.19- 16.05 | 0.45 | 2.25- 15.32 | 0.40 | 1.46- 9.6 | 0.41 | 2.28- 26.61 | 0.50 | 2.01- 11.92 | 0.43 | 2.67- 30.16 | 0.52 | 2.05- 13.37 | 0.43 |
| Hypoxanthine | 102- 253 | 0.19 | 141- 264 | 0.15 | 129- 232 | 0.13 | 88 -174 | 0.12 | 119- 245 | 0.16 | 150- 242 | 0.13 | 118- 257 | 0.15 | 80- 162 | 0.17 |
| Isobutyric acid | 3.08 -14.8 | 0.32 | 3.15 -19.5 | 0.45 | 2.66- 13.99 | 0.40 | 2.75 -12.62 | 0.35 | 3.08- 15.21 | 0.31 | 3.27- 15.81 | 0.36 | 3.25- 14.36 | 0.33 | 1.56- 10.21 | 0.23 |
| L-alanine | 354- 1835 | 0.32 | 241- 1376 | 0.21 | 518- 1526 | 0.18 | 216- 1723 | 0.23 | 187- 1171 | 0.23 | 480- 1334 | 0.21 | 482- 1601 | 0.21 | 521- 1715 | 0.25 |
| L-alpha-aminobutyric acid | 6.24- 105.15 | 0.46 | 11.58- 102.93 | 0.63 | 7.99- 99.03 | 0.53 | 4.31- 64.04 | 0.56 | 3.79- 92.07 | 0.79 | 4.36 -88.44 | 0.87 | 6.35- 82.56 | 0.65 | 6.44- 94.43 | 0.66 |
| L-asparagine | 20.68- 112.43 | 0.32 | 9.21- 75.33 | 0.38 | 24.94- 85.72 | 0.29 | 19.75- 87.81 | 0.28 | 20.39- 62.64 | 0.26 | 6.8- 88.47 | 0.36 | 20.01- 81.4 | 0.35 | 32.33- 93.86 | 0.26 |
| L-aspartate | 10.04- 62.16 | 0.37 | 10.75- 39.05 | 0.25 | 14.27- 48.08 | 0.25 | 11.27- 60.8 | 0.29 | 8.84- 34.88 | 0.28 | 8.86- 33.81 | 0.27 | 7.8- 40.84 | 0.33 | 17.34- 41.38 | 0.18 |
| L-glutamine | 175 -602 | 0.24 | 94 -599 | 0.32 | 218- 679 | 0.23 | 187- 718 | 0.25 | 161- 588 | 0.27 | 128- 512 | 0.30 | 142- 543 | 0.31 | 254- 738 | 0.25 |
| L-glutamic acid | 258- 875 | 0.28 | 136- 644 | 0.30 | 168- 764 | 0.23 | 99- 897 | 0.34 | 128- 548 | 0.25 | 135- 596 | 0.27 | 169- 728 | 0.28 | 122- 572 | 0.20 |
| L-glycine | 772- 2436 | 0.27 | 337- 1929 | 0.26 | 544- 2274 | 0.24 | 466- 2084 | 0.21 | 493- 2542 | 0.31 | 631-1896 | 0.24 | 519- 1662 | 0.25 | 809- 2674 | 0.26 |
| L-histidine | 6.61- 69.3 | 0.51 | 5.9- 65.17 | 0.64 | 5.7-54.36 | 0.54 | 7.15-50.5 | 0.39 | 9.01-50.18 | 0.41 | 3.66-62.61 | 0.45 | 7.79-63.98 | 0.57 | 7.32-51.83 | 0.37 |
| L-isoleucine | 32.66-197.6 | 0.33 | 17.93-176.69 | 0.51 | 17.73-103.97 | 0.44 | 13.39-89 | 0.48 | 13.69-160.72 | 0.67 | 13.46-131.88 | 0.52 | 14.04-107.4 | 0.52 | 26.17-118.74 | 0.36 |
| L-Lactic acid | 5081- 19336 | 0.29 | 4645- 16686 | 0.22 | 5376- 16705 | 0.25 | 4786 -15468 | 0.27 | 6581- 16285 | 0.17 | 6045- 15662 | 0.18 | 5727- 20166 | 0.26 | 3592- 12914 | 0.25 |
| L-lysine | 79-551 | 0.32 | 138-402 | 0.25 | 85-456 | 0.30 | 112-444 | 0.30 | 93-316 | 0.27 | 77-348 | 0.23 | 126-409 | 0.24 | 45- 311 | 0.32 |
| L-leucine | 55-227 | 0.28 | 61-220 | 0.28 | 71-173 | 0.23 | 51-151 | 0.23 | 41-226 | 0.28 | 53-156 | 0.20 | 35-201 | 0.26 | 65-199 | 0.23 |
| L-methionine | 6.94-95.03 | 0.50 | 7.93-116.25 | 0.47 | 16.36-101.6 | 0.38 | 7.87-69.4 | 0.44 | 4.49-61.77 | 0.53 | 8.62-58.6 | 0.44 | 8.81-85.16 | 0.52 | 10.52-85.84 | 0.38 |
| L-ornithine | 32-210 | 0.41 | 30-221 | 0.43 | 51-245 | 0.48 | 22-163 | 0.39 | 19-128 | 0.43 | 20-174 | 0.37 | 29-242 | 0.46 | 70-295 | 0.34 |
| L-phenylalanine | 36.31-110.36 | 0.20 | 40.56-113.32 | 0.20 | 43.39-96.29 | 0.17 | 45.28-132.13 | 0.18 | 43.07-127.2 | 0.24 | 38.62-97.58 | 0.18 | 54.07-90.03 | 0.13 | 38.9-99.88 | 0.17 |
| L-proline | 118-489 | 0.25 | 74-411 | 0.28 | 85-459 | 0.25 | 122-357 | 0.20 | 107-295 | 0.18 | 98-339 | 0.19 | 113-372 | 0.21 | 159-554 | 0.25 |
| L-serine | 97-383 | 0.28 | 65-242 | 0.22 | 101-306 | 0.24 | 77-319 | 0.21 | 63-291 | 0.28 | 90-207 | 0.17 | 89-321 | 0.26 | 103-331 | 0.23 |
| Oxoglutarate | 43.62-175.09 | 0.28 | 30.4-182.39 | 0.33 | 56.29-137.85 | 0.19 | 17.94-143.43 | 0.28 | 39.37-138.91 | 0.26 | 43.42-116-37 | 0.21 | 31.87-126.6 | 0.29 | 42.99-99.87 | 0.18 |
| Pyruvic acid | 248-619 | 0.21 | 316-590 | 0.13 | 248-486 | 0.14 | 191-427 | 0.16 | 172-487 | 0.18 | 307-550 | 0.10 | 136-485 | 0.27 | 233-519 | 0.17 |
| L-threonine | 191-860 | 0.39 | 70-703 | 0.42 | 54-805 | 0.52 | 90-1232 | 0.42 | 80-900 | 0.45 | 143-741 | 0.33 | 97-1021 | 0.55 | 66-886 | 0.42 |
| L-tyrosine | 29.53-127.93 | 0.29 | 37.1-120.63 | 0.25 | 42.13-110.82 | 0.24 | 36.99-124.39 | 0.22 | 33.74-96.55 | 0.26 | 40.19-105.1 | 0.23 | 42.4-120.45 | 0.25 | 40.29-94.56 | 0.22 |
| L-valine | 125-389 | 0.21 | 66-323 | 0.23 | 91-358 | 0.24 | 117-289 | 0.18 | 72-295 | 0.19 | 108-294 | 0.21 | 95-323 | 0.24 | 159-353 | 0.19 |

Supplementary Table S3. The minimum (min), maximum (max) and coefficient of variation (cv) of 33 heritable metabolites in batch 9-15.

| **Batch** | **9** | | **10** | | **11** | | **12** | | **13** | | **14** | | | **15** | | |
| --- | --- | --- | --- | --- | --- | --- | --- | --- | --- | --- | --- | --- | --- | --- | --- | --- |
| **Metabolite μM** | **min-max** | **CV** | **min-max** | **CV** | **min-max** | **CV** | **min-max** | **CV** | **min-max** | **CV** | **min-max** | **CV** | **min-max** | | **CV** |  |
| 2-Hydroxybutyrate | 11.32- 61.02 | 0.39 | 5.85 -75.02 | 0.53 | 7.09- 94.33 | 0.58 | 7.16- 982.22 | 3.56 | 5.8- 31.09 | 0.36 | 10.5- 523.93 | 1.75 | 6.05- 41.27 | | 0.36 |  |
| 3-Methyl-2-oxovaleric acid | 0.55- 4.11 | 0.32 | 1.3- 10.75 | 0.59 | 1.21- 9.74 | 0.54 | 1.57- 14.55 | 0.52 | 1.77- 10.44 | 0.45 | 1.74 -13.46 | 0.55 | 0.96 -5.22 | | 0.39 |  |
| Betaine | 43.7- 202.19 | 0.29 | 17.33- 167.08 | 0.38 | 11.22 -111.22 | 0.49 | 32.06- 204.19 | 0.36 | 29.75 -164.55 | 0.36 | 25.64 -188.09 | 0.41 | 46.06 -185.68 | | 0.31 |  |
| Citric acid | 73.17- 360.06 | 0.32 | 89.1- 302.86 | 0.27 | 109.77- 309.33 | 0.23 | 70.1- 342.41 | 0.26 | 121.22- 321.44 | 0.21 | 141.33- 324.95 | 0.20 | 62.3 298.77 | | 0.30 |  |
| Creatinine | 51.16- 103.7 | 0.16 | 34.74- 146.43 | 0.23 | 64.6- 115.25 | 0.12 | 54.65- 109.65 | 0.15 | 27.25- 102.71 | 0.15 | 78.38- 129.1 | 0.13 | 50.49- 111.7 | | 0.16 |  |
| D-glucose | 35-5449 | 0.45 | 998-6132 | 0.31 | 1148- 5518 | 0.23 | 10- 3774 | 0.86 | 444- 4906 | 0.42 | 538- 4897 | 0.30 | 68- 3758 | | 0.64 |  |
| Dimethylglycine | 2.71- 13.36 | 0.39 | 1.93- 34.74 | 0.59 | 1.47- 9.24 | 0.48 | 1.55- 8.03 | 0.31 | 2.29- 10.19 | 0.36 | 2.49- 22.07 | 0.49 | 2- 9.33 | | 0.32 |  |
| Hypoxanthine | 66- 183 | 0.25 | 44 -208 | 0.22 | 77 -183 | 0.22 | 120- 293 | 0.17 | 81- 271 | 0.16 | 107- 199 | 0.14 | 85- 201 | | 0.16 |  |
| Isobutyric acid | 1.65- 8.45 | 0.29 | 1.92- 10.64 | 0.33 | 3.27- 14.78 | 0.41 | 1.07- 8.9 | 0.30 | 1.94- 8.61 | 0.25 | 3.85- 13.57 | 0.26 | 2.34- 7.09 | | 0.25 |  |
| L-alanine | 456- 1074 | 0.17 | 334- 1337 | 0.25 | 567- 1364 | 0.20 | 219- 1440 | 0.27 | 549- 1823 | 0.20 | 246 -1136 | 0.21 | 421- 1102 | | 0.18 |  |
| L-alpha-aminobutyric acid | 9.57- 47.34 | 0.37 | 8.93- 89.66 | 0.57 | 6.78- 70.35 | 0.44 | 6.14- 68.56 | 0.75 | 7.35- 43.24 | 0.39 | 8.36- 100.99 | 0.64 | 5.47 50.75 | | 0.42 |  |
| L-asparagine | 29.58- 105.96 | 0.26 | 7.72- 71 | 0.31 | 17.19- 79.61 | 0.31 | 16.05- 99.37 | 0.35 | 29.77- 119.98 | 0.34 | 8.89- 100.01 | 0.33 | 30.51- 95.67 | | 0.23 |  |
| L-aspartate | 14.31- 48.37 | 0.21 | 10.69- 53.31 | 0.35 | 15.63- 45.51 | 0.21 | 4.27- 45.29 | 0.28 | 14.92- 57.75 | 0.29 | 10.59- 38.71 | 0.26 | 11.18- 47.8 | | 0.24 |  |
| L-glutamine | 259- 900 | 0.24 | 83- 628 | 0.37 | 58- 684 | 0.34 | 122- 810 | 0.37 | 61- 835 | 0.29 | 172- 626 | 0.28 | 165- 658 | | 0.24 |  |
| L-glutamic acid | 213- 682 | 0.23 | 143- 715 | 0.37 | 206-808 | 0.30 | 135-873 | 0.29 | 230-933 | 0.29 | 77- 416 | 0.38 | 143-663 | | 0.24 |  |
| L-glycine | 791- 2308 | 0.23 | 403- 2004 | 0.30 | 585-2504 | 0.30 | 333- 2626 | 0.32 | 1077- 2650 | 0.21 | 524- 2000 | 0.23 | 803- 2904 | | 0.27 |  |
| L-histidine | 6.19-57.44 | 0.48 | 8.67-47.7 | 0.34 | 7.93-46.87 | 0.36 | 2.5-42.76 | 0.56 | 1.58-41.22 | 0.66 | 7.14-64.46 | 0.30 | 7.21-44.97 | | 0.37 |  |
| L-isoleucine | 12.2-6364 | 0.37 | 9.9-127.43 | 0.60 | 15.14-92.12 | 0.59 | 16.55-186.63 | 0.51 | 15.53- 121 | 0.54 | 15.78-130.28 | 0.58 | 11.03-66.36 | | 0.41 |  |
| L-Lactic acid | 5555 -15762 | 0.23 | 3651- 17009 | 0.33 | 5264- 17362 | 0.28 | 7336- 22497 | 0.19 | 3619 -17700 | 0.25 | 3619- 17700 | 0.25 | 5862- 18811 | | 0.16 |  |
| L-lysine | 148-558 | 0.25 | 60-421 | 0.36 | 120-486 | 0.26 | 146-459 | 0.26 | 152-601 | 0.28 | 149-451 | 0.22 | 111-494 | | 0.29 |  |
| L-leucine | 64-180 | 0.21 | 28-171 | 0.26 | 60-138 | 0.19 | 39-210 | 0.28 | 42-167 | 0.23 | 71-230 | 0.21 | 67-129 | | 0.17 |  |
| L-methionine | 13.78-106.91 | 0.40 | 11.12-85.53 | 0.42 | 11.91-100.14 | 0.41 | 7.76-94.12 | 0.45 | 17.27-95.03 | 0.32 | 12.83-107.31 | 0.42 | 16.79-96.6 | | 0.39 |  |
| L-ornithine | 44-180 | 0.28 | 18-178 | 0.44 | 23-185 | 0.50 | 30-173 | 0.30 | 64-258 | 0.32 | 29-142 | 0.29 | 34-171 | | 0.36 |  |
| L-phenylalanine | 42.63-97.63 | 0.20 | 21.99-96.09 | 0.23 | 47.6-88.63 | 0.15 | 34.99-110.71 | 0.20 | 25.01-87.77 | 0.17 | 53.66-117.06 | 0.14 | 37.05-104.44 | | 0.22 |  |
| L-proline | 102-346 | 0.16 | 68-346 | 0.27 | 190-405 | 0.16 | 187-438 | 0.18 | 139-550 | 0.19 | 99-376 | 0.22 | 136-394 | | 0.17 |  |
| L-serine | 96-343 | 0.23 | 58-296 | 0.25 | 120-297 | 0.23 | 32-275 | 0.28 | 146-342 | 0.20 | 101-244 | 0.19 | 113-352 | | 0.20 |  |
| Oxoglutarate | 31.15-104.17 | 0.23 | 21.99-136.82 | 0.32 | 45.79-146.81 | 0.23 | 32.48-131.96 | 0.29 | 40.6-163.72 | 0.25 | 38.23-94.38 | 0.20 | 37.15-118.38 | | 0.22 |  |
| Pyruvic acid | 102-366 | 0.25 | 133-613 | 0.30 | 78-524 | 0.26 | 147-586 | 0.30 | 159-675 | 0.19 | 315-568 | 0.13 | 113-376 | | 0.23 |  |
| L-threonine | 166-1185 | 0.35 | 62-1108 | 0.56 | 69-694 | 0.52 | 82-748 | 0.44 | 216-956 | 0.37 | 100-628 | 0.34 | 144-1053 | | 0.37 |  |
| L-tyrosine | 36.32-140.36 | 0.25 | 20.05-116.85 | 0.32 | 30.29-105.13 | 0.29 | 35.26-92.22 | 0.22 | 32.19-126.93 | 0.24 | 35.84-135.96 | 0.23 | 26.86-92.44 | | 0.24 |  |
| L-valine | 153-348 | 0.17 | 55-256 | 0.25 | 102-347 | 0.23 | 73-382 | 0.29 | 82-285 | 0.21 | 93-262 | 0.21 | 99-238 | | 0.18 |  |
